# Effect of Trap Color on Captures of Bark- and Wood-Boring Beetles (Coleoptera; Buprestidae and Scolytinae) and Associated Predators

**DOI:** 10.3390/insects11110749

**Published:** 2020-10-30

**Authors:** Giacomo Cavaletto, Massimo Faccoli, Lorenzo Marini, Johannes Spaethe, Gianluca Magnani, Davide Rassati

**Affiliations:** 1 Department of Agronomy, Food, Natural Resources, Animals and Environment (DAFNAE), University of Padova, Viale dell’Università, 16–35020 Legnaro, Italy; massimo.faccoli@unipd.it (M.F.); lorenzo.marini@unipd.it (L.M.); 2 Department of Behavioral Physiology & Sociobiology, Biozentrum, University of Würzburg, Am Hubland, 97074 Würzburg, Germany; johannes.spaethe@uni-wuerzburg.de; 3 Via Gianfanti 6, 47521 Cesena, Italy; magnanig@tiscali.it

**Keywords:** ambrosia beetles, baited traps, bark beetles, biosecurity, checkered beetles, forest pests, insect vision, jewel beetles, surveillance

## Abstract

**Simple Summary:**

Several wood-associated insects are inadvertently introduced every year within wood-packaging materials used in international trade. These insects can cause impressive economic and ecological damage in the invaded environment. Thus, several countries use traps baited with pheromones and plant volatiles at ports of entry and surrounding natural areas to intercept incoming exotic species soon after their arrival and thereby reduce the likelihood of their establishment. In this study, we investigated the performance of eight trap colors in attracting jewel beetles and bark and ambrosia beetles to test if the trap colors currently used in survey programs worldwide are the most efficient for trapping these potential forest pests. In addition, we tested whether trap colors can be exploited to minimize inadvertent removal of their natural enemies. Our results confirmed that trap color strongly affects trapping performance. Overall, the trap colors currently adopted in most survey programs (i.e., green and black) are efficient only for certain beetle species, therefore there is a clear need to use additional trap colors to increase the range of species that can be effectively attracted. Moreover, we confirmed that many predators exploit the same visual cues as their preys, and thus trap color cannot be used as a variable to minimize bycatch.

**Abstract:**

Traps baited with attractive lures are increasingly used at entry-points and surrounding natural areas to intercept exotic wood-boring beetles accidentally introduced via international trade. Several trapping variables can affect the efficacy of this activity, including trap color. In this study, we tested whether species richness and abundance of jewel beetles (Buprestidae), bark and ambrosia beetles (Scolytinae), and their common predators (i.e., checkered beetles, Cleridae) can be modified using trap colors different to those currently used for surveillance of jewel beetles and bark and ambrosia beetles (i.e., green or black). We show that green and black traps are generally efficient, but also that many flower-visiting or dark-metallic colored jewel beetles and certain bark beetles are more attracted by other colors. In addition, we show that checkered beetles have color preferences similar to those of their Scolytinae preys, which limits using trap color to minimize their inadvertent removal. Overall, this study confirmed that understanding the color perception mechanisms in wood-boring beetles can lead to important improvements in trapping techniques and thereby increase the efficacy of surveillance programs.

## 1. Introduction

Bark and ambrosia beetles (Coleoptera: Curculionidae: Scolytinae) and jewel beetles (Coleoptera: Buprestidae) are commonly intercepted at ports of entry worldwide [1,2]. These beetles are moved internationally and domestically within fresh wood and wood-packaging materials [3,4] and can become destructive after introduction. The ambrosia beetles of the genus *Xylosandrus* [5,6] and the emerald ash borer *Agrilus planipennis* Fairmaire [7] are among the most famous recent examples, but the group of non-native bark and ambrosia beetles and jewel beetles established outside their native range includes dozens of other species [8,9]. Traps baited with pheromones and host volatiles are often used at high-risk sites and nearby natural areas to intercept incoming beetles soon after their arrival and to reduce the chances of their establishment [10,11,12]. The efficacy of this practice depends on several variables such as trap type, trap height, or lure specificity (e.g., [13,14]), which are often combined in order to attract several beetle species simultaneously (e.g., [15,16]) and hopefully minimize the inadvertent removal of associated natural enemies (e.g., [17,18,19]). Recent studies have shown that trap color can affect trap catches of longhorn beetles (Coleoptera: Cerambycidae) [20,21] but its effect on jewel beetles, bark and ambrosia beetles, and their associated beetle predators (e.g., Cleridae) has been investigated so far only for a limited number of species.

Jewel beetles largely exploit visual cues at the adult stage. Most members are diurnally active and rely largely on vision to locate both host trees where to oviposit and substrate where to feed, such as flowers in nectar- and pollen-feeders and tree crowns in tree foliage-feeders [22]. In addition, several jewel beetle adults display impressive patterns of metallic and pigmented coloration involved in mate location and recognition [23,24,25,26]. For these reasons, trap color has been highlighted as a key variable to consider when targeting jewel beetles [27]. Flower-visiting species, for example, were shown to be more attracted to traps painted with the typical flower colors, such as yellow, white, and blue, than to other trap colors such as black or green [28,29,30]. Many *Agrilus* species and other metallic-colored buprestids were instead found to be generally attracted by green and purple traps [21,31,32,33,34,35,36], even if certain species or individuals of particular sex within the same species show a clear preference for only one of these two colors [37,38,39]. Most studies testing the effect of trap color on jewel beetles have been conducted in North America after the invasion of the emerald ash borer *A. planipennis* in 2002 [40], whereas in Europe similar investigations are still rare and are mainly based on a limited number of colors [21,41,42,43].

Bark and ambrosia beetles exploit visual cues primarily to distinguish between host and non-host trees [44,45,46]. The bark of different tree species is characterized by different reflectance intensities, and this visual feature can complement odors (i.e., host volatiles) to discriminate between different tree species [47]. Supporting this hypothesis, several conifer-infesting bark and ambrosia beetles (i.e., *Dendroctonus* spp. and *Trypodendron lineatum* (Olivier)) were found to be more attracted by black-colored traps than white-colored traps, as black traps better resemble the color of their host tree bark [44,45,48,49,50]. Studies comparing several trap colors simultaneously showed a general preference of bark and ambrosia beetles for dark colors with long wavelengths (e.g., black, brown, red) over lighter colors such as white and yellow [51,52,53,54,55]. Some bark and ambrosia beetle species attacking broadleaf trees were instead found to have a distinct preference for a specific color. *Hypocryphalus mangiferae* Stebbing and *Xylosandrus crassiusculus* (Motschulsky) were more attracted by green traps or green light emitting diodes over other trap or light colors, respectively [56,57,58]; *Anisandrus dispar* (Fabricius) and *Cryphalus ruficollis* Hopkins preferred green over purple traps [16].

Natural enemies of wood-boring beetles, including beetle predators, also exploit visual cues along with tree volatiles and prey pheromones [59,60] to locate trees or other substrates (e.g., flowers) where they can find their preys. This process may imply the use of the same visual cues used by their prey for host selection. Previous studies showed, for example, that the bark beetles *Dendroctonus frontalis* Zimmermann and *Dendroctonus brevicomis* LeConte, and their major predators, *Thanasimus dubius* (Fabricius) (Cleridae) and *Temnochila chlorodia* (Mannerheim) (Coleoptera: Trogossitidae), respectively, were all disrupted by white paint on the boles of host trees, but that the pattern of disruption of the predator was very similar to that of the prey with which they are more closely associated [48,61]. In particular, *D. brevicomis* and its predator, *T. chlorodia*, were less affected by color than *D. frontalis* and *T. dubius* [48,61]. A similar pattern was also shown in some of the main natural enemies of *Ips* spp.; catches of the longlegged fly *Medetera bistriata* Parent (Diptera: Dolichopodidae) and the clown beetles *Platysoma* spp. (Coleoptera: Histeridae) were significantly lower on white-painted logs than on non-painted logs [62]. These studies indicated a preference of wood-boring beetle natural enemies for dark colors, although this was only partially confirmed in lab conditions [63,64]. However, a study comparing a wide range of trap colors simultaneously has not been carried out to date.

In this study, we investigated the effect of trap color on (i) jewel beetles, (ii) bark and ambrosia beetles, and (iii) their most common associated predators (i.e., checkered beetles, Cleridae). In particular, we tested whether species richness (i.e., number of species) and abundance (i.e., number of individuals) of these three beetle groups would vary using trap colors different to those currently used for the surveillance of jewel beetles (i.e., green [14,21]) and bark and ambrosia beetles (i.e., black [14]). To this aim, we used traps painted with eight different colors, i.e., black, brown, red, yellow, green, blue, purple and grey. We tested the effect of trap color at both family/subfamily and single species taxonomic levels. We predicted that most jewel beetles can be reliably trapped with the commonly used green traps, except for flower-visiting species, which may prefer traps painted with typical flower colors such as yellow or blue. In addition, we predicted that black traps can reliably attract bark and ambrosia beetles and associated checkered beetles, even if preferences for other dark colors may arise at the species level.

## 2. Materials and Methods

### 2.1. Study Sites

The methods, experimental design, and results regarding effects of trap color on detection of longhorn beetles have already been described [20] but basic methods are reported here for the reader’s convenience. The study was conducted at 16 sites in north-eastern Italy (Table S1 and Figure S1). Study sites were located in the Euganean Hills area, Veneto Region, which is characterized by the presence of fragmented forest patches interspersed with urban areas and crop fields. Three main forest types occur in the area: (i) chestnut (*Castanea sativa* Miller) forest, which covers approximately 15% of the area; (ii) dry forests dominated by *Fraxinus ornus* L. and *Ostrya carpinifolia* Scopoli, which cover approximately 30% of the area; and (iii) oak (*Quercus pubescens* Willd.) forests, which cover approximately 10% of the area. In addition, around 38% of the woodland is occupied by the invasive black locust, *Robinia pseudoacacia* L. Some plantations of Austrian pine (*Pinus nigra* Arnold) are also present (approximately 2%).

### 2.2. Trap Type, Trap Color and Trap Height

Hand-made panel traps were used at all 16 sites. Traps were composed of two crossed panels of corrugated plastic (height = 90 cm; width = 34.5 cm) covered on the top by a 28.5 × 28.5 cm cap, and a wide squared-funnel attached to the bottom (height = 42.5 cm; width = 35.4 cm) (Figure S1). A collector cup (height = 19 cm; width = 13 cm) was screwed on to the bottom of the funnel. We used panel traps because they are known to be efficient towards several families of wood-boring beetles [14,65]. Traps were painted with eight different colors that cover a wide range of the visible spectrum, i.e., grey, yellow, green, red, blue, brown, purple and black (corresponding to the international color standard RAL numbers 7034, 1018, 6037, 3020, 5015, 8002, 4008 and 9005, respectively) (Colorificio Seven Colors, Sant’Angelo di Piove di Sacco, Padova, Italy) (Figure S2). All traps were also coated with Fluon (10% solution in water) (Insect-A-Stop, Springwood, Australia), which improves the trapping efficacy of several wood-boring beetles, rendering the trap surface more slippery without affecting the reflectance of the coated surface [34,66]. Collector cups were half-filled with 50% solution of ethylene glycol to kill and preserve captured beetles [14]. This solution was renewed during each trap check. At each site, traps were hung along the forest edge on a steel wire with collecting cups about 5–7 m above the ground, keeping a distance of about 1.5 m among individual traps (Figure S1). The trap height was selected in order to maximize the chance of catching beetles living either in the canopy or in the understory [67,68]. Similarly, the choice to set up traps along the forest edge was taken to maximize the chance of catching beetles that are active either within the forest (i.e., non-flower visitors) or in the nearby open areas (i.e., flower-visitors). We are aware, however, that this choice of location might have led us to miss some of the species that live in the forest interior [13].

### 2.3. Experimental Scheme and Attractive Lures

The eight trap colors were tested using a randomized complete block design, in which each block was represented by a different site (*n* = 16). Traps were placed in the field in mid-May and were then checked every three weeks for a total of 5 times (i.e., 5 June, 26 June, 17 July, 7 August, 28 August 2019). All trapped jewel beetles, bark and ambrosia beetles, and checkered beetles were identified to species using morphological features and keys [69,70,71]. Jewel beetles were also divided into two categories based on their feeding habits: flower-visitors (i.e., *Anthaxia* spp., *Acmaeoderella* spp., and *Coraebus* spp.) vs. non-flower visitors (all other buprestid genera captured). Voucher specimens were deposited in the insect collection of the Entomology laboratory at the Department of Agronomy, Food, Natural Resources, Animals and Environment, University of Padua (Padova, Italy).

Each trap was baited with a multi-lure blend primarily developed for longhorn beetles [72]. The blend included 8 longhorn beetle pheromones (i.e., fuscumol (volume amount: 50 mg); fuscumol acetate (50 mg); geranyl acetone (25 mg); 3-hydroxyhexan-2-one (50 mg); prionic acid (1 mg); 2-methylbutan-1-ol (50 mg); anti-2,3-hexanediol (50 mg); monochamol (50 mg)) and UHR (= ultra-high release) ethanol. This kind of multi-lure blend is commonly used for the surveillance of longhorn beetles [72] but reliably attracts many jewel beetles and bark and ambrosia beetles [15,16,21]. The release rate of the pheromone blend determined by mass loss under 20 °C conditions was estimated to be 0.0263 ± 0.002 g/d, whereas that of UHR ethanol was around 1.5 g/d [72]. All pheromones were purchased from ChemTica Internacional, S.A. (Heredia, Costa Rica) except prionic acid, which was produced by Alpha Scents Inc. (West Linn, Oregon, USA). UHR ethanol was purchased from the Spanish company Econex. Pheromones were dissolved in isopropanol as a carrier to a total volume of 1 mL per lure. These one-ml aliquots were filled in glass vials with screw caps and stored at 4 °C until used. At the beginning of the trapping trial and at each trap check (i.e, every three weeks), the content of glass vials was emptied into a clear polyethylene sachet (Minigrip, 4 cm x 6 cm; Dutscher, Brumath, France) containing a cotton cylinder, which was hung in the center of the trap using a string. The ethanol was released by a polylaminated aluminum-foil “blister” that was hung in the center of the trap directly below the sachet containing the pheromone blend. Ethanol was replaced only once during the trapping season based on its expected field life (i.e., 60 days).

### 2.4. Statistical Analysis

The effects of trap color (categorical variable) on species richness and abundance of jewel beetles, bark and ambrosia beetles and checkered beetles was tested using generalized linear-effect models (GLMMs). The total number of species (i.e., species richness) and individuals (i.e., abundance) collected per each trap color over the entire trapping period were the dependent variables. The site was included in the model as a random factor to account for spatial dependence in the sampling design. Models were fitted with the function “glmer.nb” in the package “lme4” [73] available for R [74]. The same approach was used to test the effect of the trap color on the abundance of single species. In this case, the number of individuals collected per trap color over the entire trapping season (negative binomial distribution) was included in the model as a dependent variable and the site as a random factor. We analyzed only species represented by at least 30 individuals throughout the trapping season (Table 1). In addition, because of issues with heterogeneity in variances, any color or site with a zero total catch for a given species was omitted from the analysis [75]. In all cases, model overdispersion and residual distribution were checked through the “DHARMa” package [76]. In all analyses, we used planned contrasts between black or green (control) and the other colors instead of a post-hoc multiple comparison test between all the color combinations. We planned this analysis a priori considering the number of replicates (n. of sites) according to the power of this analysis. The large number of pairwise comparisons (*n* = 28) would have required a much higher number of replicates that were not technically feasible.

## 3. Results

### 3.1. General Results

A total of 16,815 individuals, representing 53 beetle species were captured (Table 1). The buprestids were the most species-rich group (33 species), followed by bark and ambrosia beetles (16 species) and checkered beetles (4 species). By contrast, most individuals were bark and ambrosia beetles, followed by checkered beetles and jewel beetles (Table 1). Among the jewel beetles, 10 species were classified as flower visitors, whereas the remaining 23 species were classified as foliage feeders. Most flower-visiting species belonged to the genus *Anthaxia*; *Anthaxia thalassophila* (Abeille) was the most abundant species, whereas 6 species were represented by less than 10 individuals each (Table 1). Among the non-flower visitors, more than half of trapped species belonged to the genus *Agrilus*; *Agrilus hastulifer* (Ratzeburg) was the most abundant species, followed by *Agrilus graminis* Kiesenwetter and *Lamprodila mirifica* (Mulsant). All jewel beetles were native species. Among bark and ambrosia beetles, the native *Xyleborinus saxesenii* (Ratzeburg) and the exotic *Xylosandrus crassiusculus* were by far the most abundant species. Among the checkered beetles, *Clerus mutillarius* F. was the most abundant species, followed by *Thanasimus formicarius* (L.). Both of these clerids are common predators of bark and ambrosia beetles [77].

### 3.2. Effect of Trap Color on Jewel Beetles

**Figure 1:**
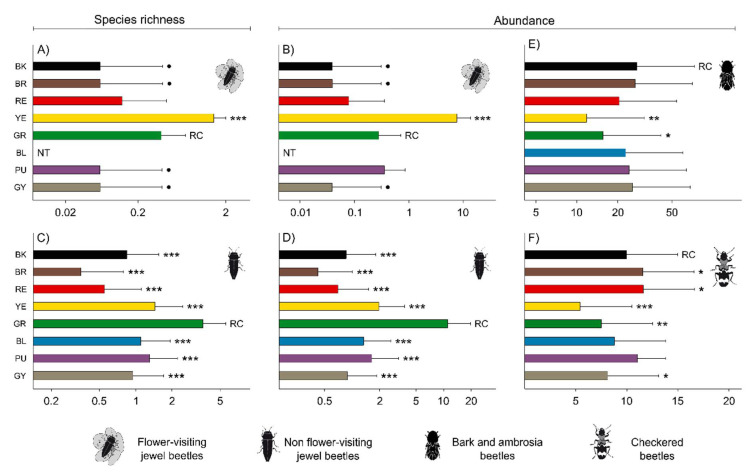
Effect of trap color on species richness and abundance of flower-visiting jewel beetles (**A**–**B**) and non-flower visiting jewel beetles (**C**–**D**), and abundance of bark and ambrosia beetles (**E**) and checkered beetles (**F**), based on the glmm.nb model output. Within each panel, colors that attracted a significantly different mean number of species or individuals than green traps (reference color for jewel beetles) and black traps (reference color for bark and ambrosia beetles and checkered beetles) are indicated with black asterisk/s or black circle depending on the *p*-value: *** = *p* < 0.001; ** = *p* < 0.01; * *p* < 0.05; ● = *p* < 0.1. Error bars indicate the 95% confidence intervals for the mean. NT = not tested colors. RC = reference color. Only variables significantly affected by trap color are displayed.

**Figure 2:**
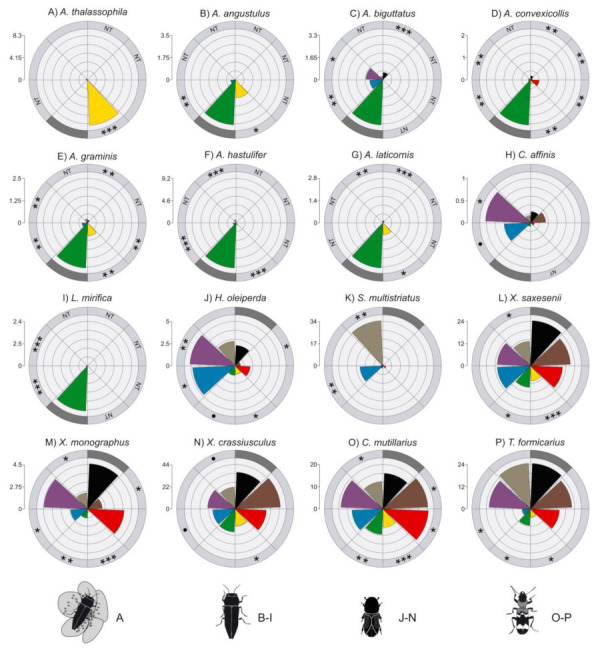
Radar plots showing the proportion (obtained from the glmm.nb model output) of flower-visiting jewel beetle (**A**), non-flower visiting jewel beetle (**B**–**I**), bark and ambrosia beetle (**J**–**N**), and checkered beetle (**O**–**P**) individuals collected in traps painted with the eight tested colors. Within each radar plot, trap colors are displayed clockwise as follows: black, brown, red, yellow, green, blue, purple and grey. Data are shown only for species that were significantly affected by trap color (Table S2). Within each radar plot, colors that performed significantly better than green for buprestids and black for bark and ambrosia beetles and checkered beetles are indicated with black asterisk/s or a black circle depending on the *p*-value: **** = *p* < 0.001; ** = *p* < 0.01; * = *p* < 0.05; ● = *p* < 0.1. Reference color (i.e, green or black) are indicated with dark grey in the outer ring. NT = not tested colors.

Both species richness and abundance of flower-visiting and non-flower-visiting jewel beetles were significantly affected by trap color (Table S2). For flower-visiting species, both the mean number of trapped species and the mean number of trapped individuals were significantly higher in yellow traps than in green traps, whereas green traps performed better than grey, black and brown traps (Table S3; Figure 1A,B). For non-flower visiting buprestids, both the mean number of trapped species and the mean number of trapped individuals were significantly higher in green traps than all the other tested colors (Table S3; Figure 1C,D). At the species level, we were able to test the effect of trap color on only one flower-visiting species and on eight non-flower-visiting species (Table S2). The flower-visitor *A. thalassophila* was caught significantly more in yellow traps than in green traps, whereas no difference was observed between green traps and purple, blue, and red traps (Table S3 and Figure 2A). Green traps outperformed the other tested trap colors in the non-flower-visiting buprestids *Agrilus angustulus* (Illiger) (Figure 2B), *Agrilus biguttatus* (Fabricius) (Figure 2C), *Agrilus convexicollis* Redtenbacher (Figure 2D), *A. graminis* (Figure 2E), *A. hastulifer* (Figure 2F), *Agrilus laticornis* (Illiger) (Figure 2G) and *L. mirifica* (Figure 2I) (Table S2), whereas purple and blue traps performed significantly better than green traps for *Chrysobothris affinis* (Fabricius) (Figure 2H).

### 3.3. Effect of Trap Color on Bark Beetles, Ambrosia Beetles and Checkered Beetles

Trap color affected the abundance but not the species richness of bark and ambrosia beetles (Table S2). For abundance, black traps caught a significantly higher number of individuals than both yellow and green traps but performed similarly to the other tested colors (Table S4 and Figure 1E). At the species level, we were able to test the effect of trap color on ten scolytine species, four bark beetles and six ambrosia beetles (Table S2). Among the bark beetles, *Hylesinus oleiperda* (Fabricius) individuals were caught significantly more by purple and blue traps than by black traps (Figure 2J), and *Scolytus multistriatus* Marsham individuals were caught significantly more by grey and blue traps than black traps (Figure 2K). For *H. oleiperda*, however, black traps performed significantly better than green and yellow traps (Figure 2J). *Scolytus rugulosus* Müller and *Scolytus intricatus* (Ratzeburg) were instead not affected by trap color (Table S2). Black traps performed generally well for ambrosia beetles. *X. saxesenii* individuals were caught significantly more in black traps than green, yellow and grey traps (Figure 2L), but trap catches did not differ between black traps and purple, blue, red and brown traps. *Xyleborus monographus* (Fabricius) individuals were caught significantly more by black traps than grey, brown, yellow, blue and green, but performed similarly to purple and red traps (Figure 2M). Black traps performed significantly better than yellow, blue and grey traps for *X. crassiusculus* (Figure 2N). No effect of trap color was found for *A. dispar, Xyleborus dryographus* (Ratzeburg) and *Xylosandrus germanus* (Blandford) (Table S2).

Trap color significantly affected abundance but not species richness of checkered beetles (Table S2). Black traps caught significantly more individuals than yellow, grey and green traps but were outperformed by both red and brown traps (Table S4 and Figure 1F). At the species level, we were able to test the effect of trap color on two out of the three trapped clerid species (Table S2). *C. mutillarius* was caught significantly more by red and brown traps than by black traps, but black traps caught more individuals than yellow, green and grey traps (Table S4 and Figure 2O). Similarly, for *T. formicarius*, black traps performed better than yellow, blue and green traps but did not differ from the other tested colors (Table S4 and Figure 2P).

## 4. Discussion

Baited traps set up at ports of entry are used in surveillance programs to target wood-boring beetles potentially arriving via timber and wood products used in international and national trade [78]. Selecting the best trap color for the targeted species or groups of species is essential to improve the chances of early detection [16,20,21]. Here we showed that the trap colors currently used for the surveillance of jewel beetles (i.e., green) [14,21] and bark and ambrosia beetles (i.e., black) [14] can be generally considered efficient, but we also highlighted that the use of more trap colors would increase the pool of species that can be trapped. Furthermore, we confirmed that checkered beetles have color preferences similar to those of their prey (i.e., bark and ambrosia beetles) [48,61], and this limits the usefulness of particular trap colors to minimize the inadvertent bycatch of clerids. Finally, the species-specific color preferences that we found for certain jewel beetles and bark and ambrosia beetles indicated that understanding color perception mechanisms of wood-boring beetles can lead to important improvements in trapping techniques, with important positive impacts on the overall efficacy of surveillance programs.

Trap color significantly affected species richness and the abundance of jewel beetles, although with mixed responses depending on their feeding habits. Flower-visiting jewel beetles were caught significantly more often with yellow traps than with green traps, whereas non-flower visiting species were caught significantly more frequently by green traps than the other tested colors. The preference of pollen- and nectar-feeding species for the typical flower colors is well documented in insects [20,79,80], including jewel beetles [28,30]. Species belonging to the genera *Anthaxia* and *Acmaeoderella*, in particular, were already found to consistently prefer yellow and white traps over other trap colors [28,29,30], and our results confirmed this pattern. The preference of non-flower visiting jewel beetle species for green traps was also expected. *Agrilus* spp. and other metallic-colored buprestid species have a complex set of photoreceptors that allow them to rely on color vision for mate finding [23,81]. Females commonly rest on tree foliage and males locate them, guided by the spectral emission profiles of the females’ body [23,24,25]. For this reason, metallic green-colored species, such as *Agrilus* spp., are attracted by green traps, e.g., [31,41,42,43,82], whereas species with darker metallic coloration such as *C. affinis* (in our study) or *Coroebus undatus* (F.) (in previous studies) are mainly attracted by darker trap colors, such as purple or blue [35,36,81].

In addition, trap color significantly affected abundance but not species richness of bark and ambrosia beetles, mostly confirming patterns observed in previous studies, i.e., black-colored traps are generally more attractive to bark and ambrosia beetles than yellow or light-colored traps [44,45,48,51,52,53,54,55]. This pattern is related to wood-boring beetle host selection. That is, choosing the darkest color might be a proper mechanism to distinguish a dark object (a tree trunk) against a bright background (the sky), and thus to locate host plants throughout the landscape. This general pattern is however not valid for all bark and ambrosia beetles, as some species show species-specific preferences [16,56,57,58]. We found, for example, that *H. oleiperda* and *S. multistriatus* were more attracted by purple/blue and grey/blue traps, respectively, than black traps. Such preferences might be linked to the bark features of their most commonly colonized host trees. The bark of different tree species is characterized by different reflectance intensity, which might be used by adult beetles along with host volatiles to distinguish between host vs non-host trees [47], although random landing can occur [83]. *Hylesinus oleiperda* and *S. multistriatus* mainly attack broadleaf trees with greyish or light-colored tree bark (i.e., olive and elm, respectively), and this might explain their attraction to grey or blue traps. By contrast, polyphagous species can be expected to rely more on generic volatiles than bark features for host selection. The lack of marked preferences between black and the other dark colors that we found for *X. saxesenii*, *X. crassiusculus* or *X. germanus*, which are known to colonize hundreds of tree species locating them using ethanol as an olfactory cue [84], might support this hypothesis.

Similar to what we observed for bark and ambrosia beetles, trap color significantly affected checkered beetle abundance but not species richness, with black generally preferred over light colors, and yellow and green traps mostly avoided. These patterns suggest that predators (i.e., checkered beetles) use similar visual cues as their prey (i.e., bark and ambrosia beetles) [48,63], with dark colors exploited along with olfactory cues to locate host trees and their scolytine prey. Nonetheless, we did observe some differences at the species level, with *C. mutillarius* showing a preference for brown and red over black, whereas black traps attracted *T. formicarius* equally to brown, grey, red and purple traps. These differences may be linked to differences in the range of their prey, i.e., *T. formicarius* is known to prey on a wide range of bark and ambrosia beetle species that colonize both conifer and broadleaf trees, whereas *C. mutillarius* preys only on bark beetle species colonizing conifers [77]. Therefore *T. formicarius* may rely less on specific visual cues (i.e., bark characteristics to locate trees and thus their prey) than the more prey-specific *C. mutillarius*. Further evidence is, however, needed to confirm this trend.

## 5. Conclusions

Our study confirmed that trap color is a key trapping variable to consider when planning surveillance programs for jewel beetles and bark and ambrosia beetles. For jewel beetles, green traps, which are commonly recommended for biosecurity surveys [14,21], can be considered an efficient tool for metallic green-colored species, especially *Agrilus* spp., but more colors, especially yellow and purple, should be used to increase the chances of detecting flower-visiting buprestid species and dark-metallic colored species, respectively. For bark and ambrosia beetles, black can be generally considered an efficient trap color [14], but other trap colors should be considered. As we found for *H. oleiperda* and *S. multistriatus*, monophagous or oligophagous species might have more pronounced color preferences than polyphagous species, and specific colored traps may prove to be more effective than the commonly used black traps. Our results also highlighted that trap color can partially minimize the inadvertent removal of certain checkered beetles (e.g., black traps reduce catches of *C. mutillarius*) but not others (e.g., *T. formicarius*). Thus, other strategies must be developed to minimize the bycatch of predators, such as modification of trap design [18] and displacement or selective choice of lures [17,19]. Finally, given that our study was carried out only in 2019 and in a single area located in north-eastern Italy, it would be worth repeating the same trapping trial in other locations and in different years as the response of wood-boring beetles to baited traps has already been shown to potentially change in space and time, e.g., [16].

## Figures and Tables

**Table 1:** Total number of jewel beetle (Buprestidae), bark and ambrosia beetle (Scolytinae), and checkered beetle (Cleridae) individuals trapped by each trap color. Jewel beetles are divided into flower visitors and non-flower visitors. Species are listed alphabetically within each family. An * indicates a species exotic to Italy. Color abbreviations are: BK = black; BR = brown; RE = red; YE = yellow; GR = green; BL = blue; PU = purple; GY = grey.

Buprestidae	BK	BR	RE	YE	GR	BL	PU	GY	Total
Flower visitors									
*Acmaeoderella flavofasciata*	-	-	-	3	-	-	-	-	3
*Anthaxia cichorii*	-	-	-	2	-	-	-	-	2
*Anthaxia croesus*	1	-	-	12	-	-	-	-	13
*Anthaxia fulgurans*	-	-	-	1	-	-	-	-	1
*Anthaxia hungarica*	-	-	-	1	-	-	-	-	1
*Anthaxia manca*	-	-	-	1	2	-	-	-	3
*Anthaxia millefolii polychloros*	-	-	-	9	1	-	-	-	10
*Anthaxia nitidula*	-	-	-	19	-	-	-	-	19
*Anthaxia thalassophila*	-	-	1	150	3	-	9	1	164
*Coraebus elatus*	-	1	1	2	1	-	-	-	5
Total	1	1	2	200	7	0	9	1	221
Non flower visitors									
*Agrilus angustulus*	-	-	-	9	21	2	-	-	32
*Agrilus ater*	1	-	-	-	-	-	-	-	1
*Agrilus auricollis*	-	-	1	2	9	1	1	1	15
*Agrilus biguttatus*	5	-	-	-	26	7	9	2	49
*Agrilus convexicollis*	2	1	4	2	21	1	1	-	32
*Agrilus derasofasciatus*	-	-	-	5	1	-	1	-	7
*Agrilus graminis*	2	-	-	11	49	4	1	4	71
*Agrilus hastulifer*	-	-	-	3	62	4	-	4	73
*Agrilus laticornis*	2	-	-	17	40	-	-	1	60
*Agrilus obscuricollis*	-	-	1	1	2	-	-	1	5
*Agrilus olivicolor*	-	-	-	1	1	-	-	-	2
*Agrilus roscidus*	1	1	2	1	2	3	2	2	14
*Agrilus sulcicollis*	-	-	-	-	2	-	-	1	3
*Agrilus viridicaerulans rubi*	-	-	-	-	1	-	-	1	2
*Buprestis haemorrhoidalis*	-	-	-	-	-	-	1	-	1
*Buprestis novemmaculata*	-	-	-	-	-	-	2	-	2
*Capnodis tenebrionis*	1	-	-	-	-	-	-	-	1
*Chrysobothris affinis*	3	4	1	-	1	7	12	2	30
*Dicerca aenea*	2	3	3	-	-	1	3	2	14
*Dicerca berolinensis*	1	-	-	-	-	-	1	-	2
*Lamprodila mirifica*	-	-	-	-	65	2	1	-	68
*Meliboeus fulgidicollis*	-	1	4	1	5	-	-	-	11
*Trachypteris picta decostigma*	-	-	-	-	-	-	1	-	1
Total	20	10	16	53	308	32	36	21	496
**Scolytinae**									
*Ambrosiophilus atratus* *	1	-	-	-	-	2	-	-	3
*Anisandrus dispar*	11	15	10	7	5	10	12	10	80
*Cyrtogenius luteus* *	2	1	-	1	1	1	3	-	9
*Dryocoetes villosus*	-	-	-	-	-	1	-	-	1
*Hylesinus oleiperda*	16	6	12	7	8	32	37	22	140
*Orthotomicus erosus*	-	-	-	-	-	1	-	1	2
*Scolytus intricatus*	7	9	14	9	3	2	36		80
*Scolytus multistriatus*	3	1	6	4	4	37	5	69	129
*Scolytus pygmaeus*	-	4	1	1	2	3	-	2	13
*Scolytus rugulosus*	8	4	4	23	11	10	23	12	95
*Taphrorychus hirtellus*	1	-	1	-	-	-	1	-	3
*Xyleborinus saxesenii*	1177	1408	804	434	678	1086	734	696	7017
*Xyleborus dryographus*	24	4	11	6	6	11	13	9	84
*Xyleborus monographus*	27	12	20	2	6	10	24	9	110
*Xylosandrus crassiusculus* *	665	1270	610	264	541	697	420	353	4820
Xylosandrus germanus *	4	20	8	16	4	19	20	16	107
Total	1946	2754	1501	774	1269	1922	1328	1199	12693
**Cleridae**									
*Clerus mutillarius*	467	553	520	228	312	379	544	329	3332
*Denops albofasciatus*	-	-	-	2	1	-	-	-	3
*Thanasimus formicarius*	12	11	7	2	4	3	10	11	60
*Tilloidea unifasciata*	-	2	2	5	1	-	-	-	10
Total	479	566	529	237	318	382	554	340	3405

## Supplementary Materials

The following are available online at https://www.mdpi.com/2075-4450/11/11/749/s1, Figure S1: location at national and local scale of the 16 sites where the trapping study was carried out and example of how traps were set up at each site; Figure S2: spectral reflectance of traps painted with the eight different colors [85]; Table S1: location, geographic coordinates, elevation and exposure of the 16 sites located in the Euganean Hills area (Veneto region, Italy) where the trapping study was carried out in 2019; Table S2: mean (± standard error) number of species and individuals trapped by each trap color for jewel beetles, bark and ambrosia beetles and checkered beetles, and results of the GLMMs; Table S3: results of the GLMMs (i.e., contrasts vs green) testing the effect of trap color on flower-visiting and non-flower visiting jewel beetles. Table S4: results of the GLMMs (i.e., contrasts vs black) testing the effect of trap color on bark and ambrosia (Scolytinae) beetles and checkered beetles (Cleridae).
