# Supplementary material for: Effect of Trap Color on Captures of Bark- and Wood-Boring Beetles (Coleoptera; Buprestidae and Scolytinae) and Associated Predators
Source: Insects. 2020 Oct 30;11(11):749. doi: 10.3390/insects11110749 (PMC7694114; doi:10.3390/insects11110749)

**Figure S1:** location at national and local scale of the 16 sites (red dots) where the trapping study was carried out and example of how traps were set up at each site.


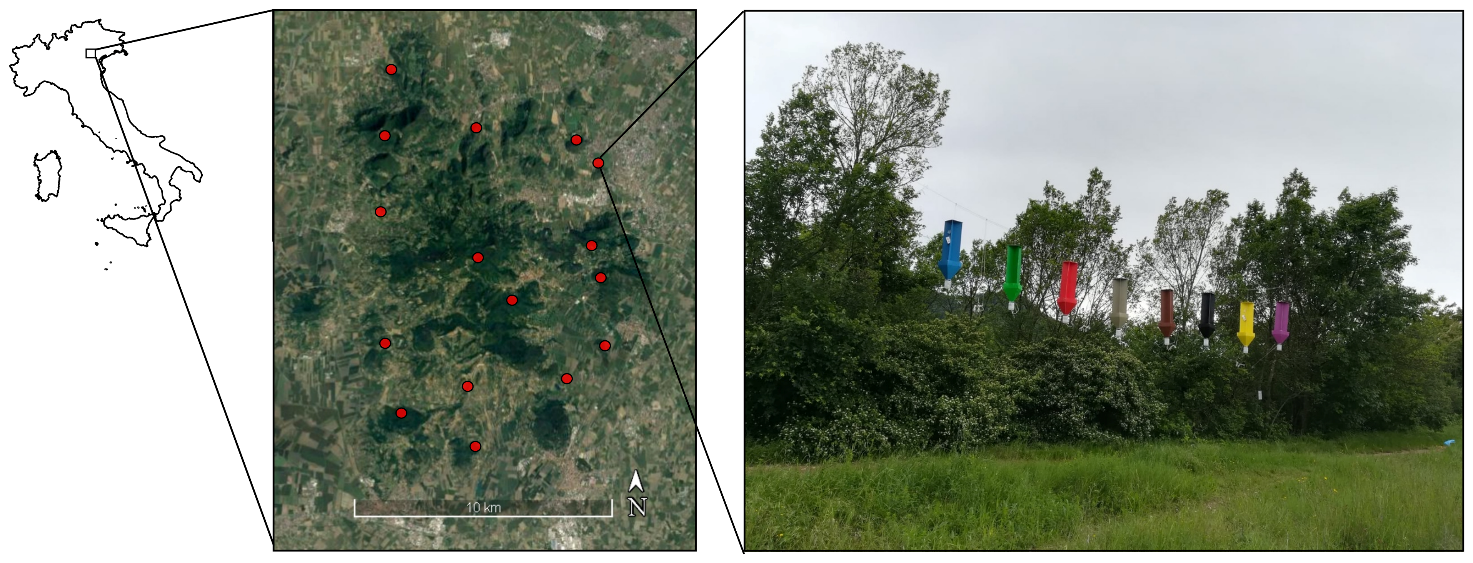

Supplement: Supplementary file 1 [file insects-11-00749-s001.zip › Compressed_Supplementary_files/Figure_S1.docx]
