# Supplementary material for: Effect of Trap Color on Captures of Bark- and Wood-Boring Beetles (Coleoptera; Buprestidae and Scolytinae) and Associated Predators
Source: Insects. 2020 Oct 30;11(11):749. doi: 10.3390/insects11110749 (PMC7694114; doi:10.3390/insects11110749)

**Figure S2.** spectral reflectance of traps painted with the eight different colors. Reflection was measured with an Ocean Optics JAZ spectrometer equipped with a pulsed xenon light source (JAZ-PX, Ocean Optics B.V., Duiven, The Netherlands) and calibrated against a white diffuse reflection standard (WS-1-SL, Ocean Optics) following standard protocols [85].


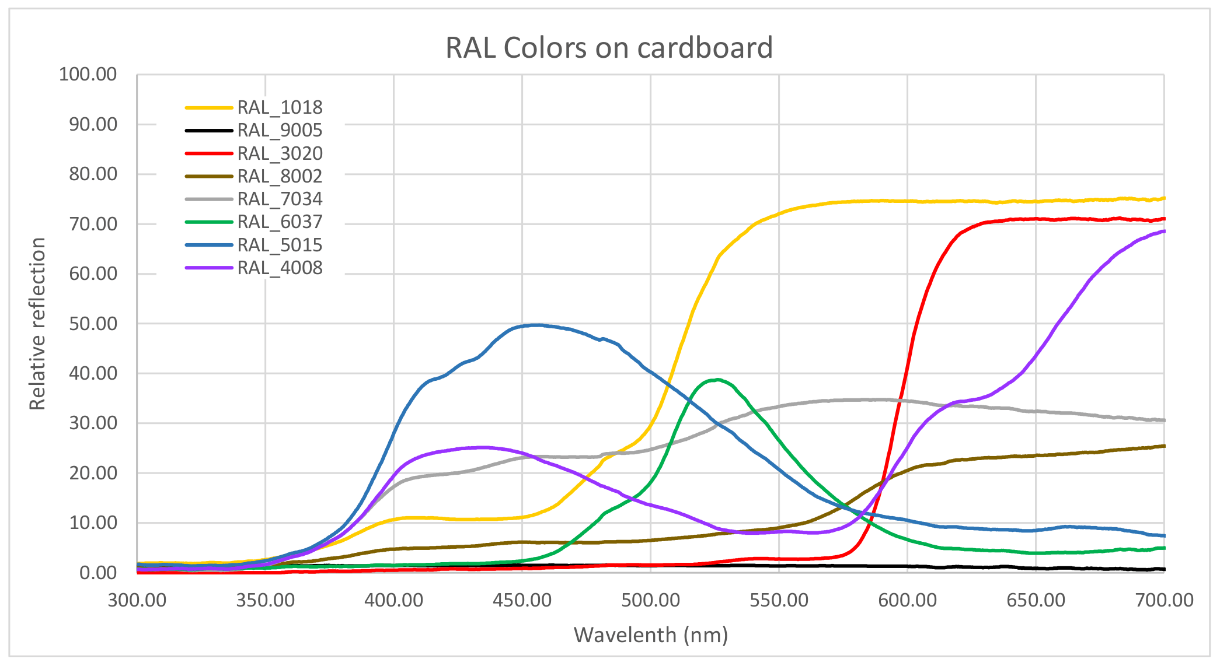

Supplement: Supplementary file 1 [file insects-11-00749-s001.zip › Compressed_Supplementary_files/Figure_S2.docx]
