# Supplementary material for: Effect of Trap Color on Captures of Bark- and Wood-Boring Beetles (Coleoptera; Buprestidae and Scolytinae) and Associated Predators
Source: Insects. 2020 Oct 30;11(11):749. doi: 10.3390/insects11110749 (PMC7694114; doi:10.3390/insects11110749)
Supplement: Supplementary file 1 [file insects-11-00749-s001.zip › Compressed_Supplementary_files/Table_S1.docx]

**Table S1:** location, geographic coordinates, elevation and exposure of the 16 sites located in the Euganean Hills area (Veneto region, Italy) where the trapping study was carried out in 2019.

| **Location** | **Latitude N** | **Longitude E** | **Elevation (m a.s.l.)** | **Exposure** |
| --- | --- | --- | --- | --- |
| Calaone (Baone) | 45°15'31.42" | 11°40'05.50" | 166 | W |
| Boccon (Vo’) | 45°19'38.27" | 11°39'09.83" | 72 | S |
| Arquà Petrarca | 45°16'20.25" | 11°44'43.24" | 31 | E |
| Roccolo (Galzignano Terme) | 45°18'55.13" | 11°42'16.00" | 257 | SE |
| Valle San Giorgio (Baone) | 45°16'10.63" | 11°41'56.58" | 71 | SW |
| Baone | 45°14'45.98" | 11°42'07.34" | 95 | SE |
| San Daniele (Abano Terme) | 45°20'37.83" | 11°46'00.38" | 50 | SE |
| Monteortone (Abano Terme) | 45°21'20.90" | 11°45'17.08" | 18 | N |
| Teolo | 45°21'19.66" | 11°39'25.05" | 410 | SE |
| Via Oslavia (Galzignano) Terme | 45°18'28.33" | 11°45'58.97" | 139 | S |
| Turri (Montegrotto Terme) | 45°19'05.07" | 11°46'23.55" | 154 | S |
| Cinto Euganeo | 45°16'58.78" | 11°39'33.75" | 111 | SE |
| Rovolon | 45°22'40.63" | 11°39'33.50" | 54 | S |
| Treponti (Teolo) | 45°21'33.22" | 11°42'07.34" | 67 | W |
| Battaglia Terme | 45°16'54.82" | 11°46'00.42" | 22 | E |
| Calaone (Baone) | 45°18'01.21" | 11°43'17.76" | 76 | N |
