# Supplementary material for: Effect of Trap Color on Captures of Bark- and Wood-Boring Beetles (Coleoptera; Buprestidae and Scolytinae) and Associated Predators
Source: Insects. 2020 Oct 30;11(11):749. doi: 10.3390/insects11110749 (PMC7694114; doi:10.3390/insects11110749)
Supplement: Supplementary file 1 [file insects-11-00749-s001.zip › Compressed_Supplementary_files/Table_S2.docx]

**Table S2**. mean (± standard error) number of species and individuals trapped by each trap color for jewel beetles, bark and ambrosia beetles and checkered beetles. Results of the GLMMs (χ2 and P value) used to test the effect of trap color are also reported. Individual species are listed in alphabetical order within each beetle group. Only species represented by at least 30 individuals were analyzed (see also Table 1). p-value: *** = P<0.001; ** = P<0.01; * P<0.05; ● = P<0.1; ns = not significant. nt = not tested.

|  | Black | Brown | Red | Yellow | Green | Blue | Purple | Grey | *χ*^2^ | *P* |  |
| --- | --- | --- | --- | --- | --- | --- | --- | --- | --- | --- | --- |
| **Bupretidae** |  |  |  |  |  |  |  |  |  |  |  |
| Flower visitors |  |  |  |  |  |  |  |  |  |  |  |
| Richness | 0.06±0.06 | 0.06±0.06 | 0.13±0.09 | 2.31±0.36 | 0.44±0.16 | 0.00±0.00 | 0.06±0.06 | 0.06±0.06 | 68.775 | >0.001 | *** |
| Abundance | 0.06±0.06 | 0.06±0.06 | 0.13±0.09 | 12.50±3.65 | 0.44±0.16 | 0.00±0.00 | 0.56±0.56 | 0.06±0.06 | 229.96 | >0.001 | *** |
| *Anthaxia thalassophila* (Abeille) | 0.00±0.00 | 0.00±0.00 | 0.06±0.06 | 9.38±3.74 | 0.19±0.10 | 0.00±0.00 | 0.56±0.56 | 0.06±0.06 | 49.189 | >0.001 | *** |
| Non-flower visitors |  |  |  |  |  |  |  |  |  |  |  |
| Richness | 1.06±0.27 | 0.44±0.13 | 0.69±0.20 | 1.81±0.46 | 4.50±0.77 | 1.38±0.29 | 1.63±0.33 | 1.19±0.31 | 97.028 | >0.001 | *** |
| Abundance | 1.25±0.32 | 0.63±0.22 | 1.00±0.40 | 3.31±1.60 | 19.25±6.29 | 2.00±0.52 | 2.25±0.49 | 1.31±0.39 | 165.7 | >0.001 | *** |
| *Agrilus angustulus* (Illiger) | 0.00±0.00 | 0.00±0.00 | 0.00±0.00 | 0.56±0.26 | 1.31±0.64 | 0.13±0.13 | 0.00±0.00 | 0.00±0.00 | 12.382 | 0.002 | ** |
| *Agrilus biguttatus* (Fabricius) | 0.31±0.18 | 0.00±0.00 | 0.00±0.00 | 0.00±0.00 | 1.63±0.74 | 0.44±0.20 | 0.56±0.27 | 0.13±0.13 | 26.245 | >0.001 | *** |
| *Agrilus convexicollis* Redtenbacher | 0.13±0.09 | 0.06±0.06 | 0.25±0.25 | 0.13±0.09 | 1.31±0.51 | 0.06±0.06 | 0.06±0.06 | 0.00±0.00 | 42.813 | >0.001 | *** |
| *Agrilus graminis* Castelnau & Gory | 0.13±0.09 | 0.00±0.00 | 0.00±0.00 | 0.69±0.33 | 3.06±2.28 | 0.25±0.11 | 0.06±0.06 | 0.25±0.11 | 36.978 | >0.001 | *** |
| *Agrilus hastulifer* (Ratzeburg) | 0.00±0.00 | 0.00±0.00 | 0.00±0.00 | 0.19±0.10 | 3.88±1.75 | 0.25±0.19 | 0.00±0.00 | 0.25±0.14 | 66.637 | >0.001 | *** |
| *Agrilus laticornis* (Illiger) | 0.13±0.09 | 0.00±0.00 | 0.00±0.00 | 1.06±1.00 | 2.50±1.47 | 0.00±0.00 | 0.00±0.00 | 0.06±0.06 | 20.006 | >0.001 | *** |
| *Chrysobothris affinis* (Fabricius) | 0.19±0.10 | 0.25±0.14 | 0.06±0.06 | 0.00±0.00 | 0.06±0.06 | 0.44±0.20 | 0.75±0.23 | 0.13±0.09 | 17.223 | 0.008 | ** |
| *Lamprodila mirifica* (Mulsant) | 0.00±0.00 | 0.00±0.00 | 0.00±0.00 | 0.00±0.00 | 4.06±2.21 | 0.13±0.13 | 0.06±0.06 | 0.00±0.00 | 44.768 | >0.001 | *** |
| **Scolitidae** |  |  |  |  |  |  |  |  |  |  |  |
| Richness | 4.63±0.69 | 4.69±0.51 | 4.38±0.49 | 3.5±0.65 | 3.81±0.68 | 4.69±0.63 | 4.63±0.63 | 4.13±0.46 | 5.431 | 0.608 | *ns* |
| Abundance | 121.63±61.27 | 172.13±102.85 | 93.81±51.46 | 48.38±19.66 | 79.31±45.89 | 120.13±72.69 | 83±30.82 | 74.94±31.64 | 15.47 | 0.030 | * |
| *Anisandrus dispar* (Fabricius) | 0.69±0.39 | 0.94±0.44 | 0.63±0.33 | 0.44±0.22 | 0.31±0.20 | 0.63±0.27 | 0.75±0.39 | 0.63±0.27 | 5.533 | 0.595 | *ns* |
| *Hylesinus oleiperda* (Fabricius) | 1.00±0.87 | 0.38±0.26 | 0.75±0.62 | 0.44±0.38 | 0.50±0.30 | 2.00±1.68 | 2.31±1.67 | 1.38±0.99 | 47.064 | >0.001 | *** |
| *Scolytus intricatus* (Ratzeburg) | 0.44±0.32 | 0.56±0.27 | 0.88±0.56 | 0.56±0.56 | 0.19±0.10 | 0.13±0.09 | 2.25±1.86 | 0.00±0.00 | 10.517 | 0.161 | *ns* |
| *Scolytus multistriatus* Marsham | 0.19±0.10 | 0.06±0.06 | 0.38±0.26 | 0.25±0.11 | 0.25±0.19 | 2.31±1.87 | 0.31±0.20 | 4.31±4.18 | 26.260 | >0.001 | *** |
| *Scolytus rugulosus* Müller | 0.50±0.34 | 0.25±0.14 | 0.25±0.19 | 1.44±1.44 | 0.69±0.51 | 0.63±0.38 | 1.44±1.44 | 0.75±0.50 | 5.199 | 0.636 | *ns* |
| *Xyleborinus saxesenii* (Ratzeburg) | 73.56±36.85 | 88.00±46.46 | 50.25±26.11 | 27.13±11.75 | 42.38±25.19 | 67.88±39.02 | 45.88±17.49 | 43.5±19.99 | 20.012 | 0.006 | ** |
| *Xyleborus dryographus* (Ratzeburg) | 1.50±0.58 | 0.25±0.14 | 0.69±0.30 | 0.38±0.15 | 0.38±0.22 | 0.69±0.46 | 0.81±0.43 | 0.56±0.29 | 11.691 | 0.111 | *ns* |
| *Xyleborus monographus* (Fabricius) | 1.69±0.58 | 0.75±0.23 | 1.25±0.60 | 0.13±0.13 | 0.38±0.20 | 0.63±0.30 | 1.5±0.61 | 0.56±0.50 | 28.932 | >0.001 | *** |
| *Xylosandrus crassiusculus* (Motschulsky)* | 41.56±24.19 | 79.38±56.34 | 38.13±25.41 | 16.5±7.49 | 33.81±20.89 | 43.56±33.34 | 26.25±12.96 | 22.06±11.34 | 16.005 | 0.025 | * |
| *Xylosandrus germanus* (Blandford)* | 0.25±0.14 | 1.25±0.89 | 0.50±0.39 | 1.00±0.75 | 0.25±0.14 | 1.19±0.75 | 1.25±0.99 | 1.00±0.52 | 11.964 | 0.102 | *ns* |
| **Cleridae** |  |  |  |  |  |  |  |  |  |  |  |
| Richness | 1.25±0.11 | 1.44±0.18 | 1.44±0.16 | 1.38±0.18 | 1.19±0.19 | 1.06±0.11 | 1.25±0.14 | 1.25±0.11 | 1.5288 | 0.981 | *ns* |
| Abundance | 29.94±10.93 | 35.38±10.99 | 33.06±9.25 | 14.81±5.04 | 19.88±6.18 | 23.88±8 | 34.63±12.72 | 21.25±6.25 | 96.501 | >0.001 | *** |
| *Clerus mutillarius* Fabricius | 29.19±10.84 | 34.56±10.82 | 32.5±9.14 | 14.25±4.98 | 19.5±6.13 | 23.69±7.97 | 34±12.59 | 20.56±6.04 | 91.954 | >0.001 | *** |
| *Denops albofasciatus* (Charpentier) | 0.00±0.00 | 0.00±0.00 | 0.00±0.00 | 0.13±0.13 | 0.06±0.06 | 0.00±0.00 | 0.00±0.00 | 0.00±0.00 | 13.663 | 0.058 | ● |
|  |  |  |  |  |  |  |  |  |  |  |  |
