# Supplementary material for: Effect of Trap Color on Captures of Bark- and Wood-Boring Beetles (Coleoptera; Buprestidae and Scolytinae) and Associated Predators
Source: Insects. 2020 Oct 30;11(11):749. doi: 10.3390/insects11110749 (PMC7694114; doi:10.3390/insects11110749)
Supplement: Supplementary file 1 [file insects-11-00749-s001.zip › Compressed_Supplementary_files/Table_S3.docx]

**Table S3.** results of the GLMMs (i.e., contrasts vs green) testing the effect of trap color on flower-visiting and non-flower visiting jewel beetles. Model estimate (est), standard error (SE), z and P values are presented for each model. Colors that attracted a significantly different mean number of species or individuals than green traps are indicated in bold and with black asterisk/s or black circle based on the p-value: *** = P<0.001; ** = P<0.01; * P<0.05; ● = P<0.1.

| **Buprestidae** | | | | | | | | | | | | | | | | | |
| --- | --- | --- | --- | --- | --- | --- | --- | --- | --- | --- | --- | --- | --- | --- | --- | --- | --- |
| **Flower visitors** | | | | | | | | | | | | | | | | | |
|  | Species richness | | | |  |  | Abundance | | | |  |  | *Anthaxia thalassophila* | | | |  |
| Vs. Green | EST | SE | z | P |  |  | EST | SE | z | P |  |  | EST | SE | z | P |  |
| Blue | nt | nt | nt | nt |  |  | nt | nt | nt | nt |  |  |  |  |  |  |  |
| Purple | -1.946 | 1.048 | -1.857 | 0.063 | ● |  | 0.243 | 0.516 | 0.470 | 0.638 | ns |  | 0.683 | 0.952 | 0.717 | 0.473 | ns |
| Grey | -1.946 | 1.050 | -1.852 | 0.064 | ● |  | -1.958 | 1.075 | -1.822 | 0.069 | ● |  | -1.133 | 1.294 | -0.876 | 0.381 | ns |
| Black | -1.946 | 1.053 | -1.847 | 0.065 | ● |  | -1.956 | 1.075 | -1.820 | 0.069 | ● |  | nt | nt | nt | nt |  |
| Brown | -1.946 | 1.058 | -1.839 | 0.066 | ● |  | -1.954 | 1.075 | -1.818 | 0.069 | ● |  | nt | nt | nt | nt |  |
| Red | -1.253 | 0.793 | -1.579 | 0.114 | ns |  | -1.264 | 0.810 | -1.562 | 0.118 | ns |  | -1.206 | 1.295 | -0.932 | 0.352 | ns |
| Yellow | 1.665 | 0.406 | 4.100 | <0.001 | *** |  | 3.314 | 0.400 | 8.287 | <0.001 | *** |  | 3.898 | 0.808 | 4.822 | <0.001 | *** |
| **Non-flower visitors** | | | | | | | | | | | | | | | | | |
|  | Species richness | | | |  |  | Abundance | | | |  |  | *Agrilus angustulus* | | | |  |
| Vs. Green | EST | SE | z | P |  |  | EST | SE | z | P |  |  | EST | SE | z | P |  |
| Blue | -1.1856 | 0.2419 | -4.902 | <0.001 | *** |  | -2.114 | 0.287 | -7.356 | <0.001 | *** |  | -2.302 | 0.744 | -3.095 | 0.002 | ** |
| Purple | -1.0185 | 0.2266 | -4.494 | <0.001 | *** |  | -1.904 | 0.282 | -6.762 | <0.001 | *** |  | nt | nt | nt | nt |  |
| Grey | -1.3322 | 0.2549 | -5.226 | <0.001 | *** |  | -2.520 | 0.317 | -7.950 | <0.001 | *** |  | nt | nt | nt | nt |  |
| Black | -1.4434 | 0.2664 | -5.417 | <0.001 | *** |  | -2.545 | 0.321 | -7.934 | <0.001 | *** |  | nt | nt | nt | nt |  |
| Brown | -2.3306 | 0.3848 | -6.057 | <0.001 | *** |  | -3.263 | 0.392 | -8.318 | <0.001 | *** |  | nt | nt | nt | nt |  |
| Red | -1.8787 | 0.3221 | -5.833 | <0.001 | *** |  | -2.760 | 0.341 | -8.105 | <0.001 | *** |  | nt | nt | nt | nt |  |
| Yellow | -0.9093 | 0.2176 | -4.179 | <0.001 | *** |  | -1.733 | 0.268 | -6.463 | <0.001 | *** |  | -0.912 | 0.422 | -2.161 | 0.031 | * |
|  | *Agrilus_biguttatus* | | | |  |  | *Agrilus convexicollis* | | | |  |  | *Agrilus graminis* | | | |  |
| Vs. Green | EST | SE | z | P |  |  | EST | SE | z | P |  |  | EST | SE | z | P |  |
| Blue | -1.232 | 0.427 | -2.884 | 0.004 | ** |  | -3.045 | 1.012 | -3.008 | 0.003 | ** |  | -2.073 | 0.603 | -3.439 | <0.001 | *** |
| Purple | -0.981 | 0.388 | -2.529 | 0.011 | * |  | -3.045 | 1.001 | -3.041 | 0.002 | ** |  | -3.486 | 1.058 | -3.295 | <0.001 | *** |
| Grey | -2.485 | 0.724 | -3.432 | <0.001 | *** |  | nt | nt | nt | nt |  |  | -2.399 | 0.670 | -3.583 | <0.001 | *** |
| Black | -1.792 | 0.534 | -3.358 | <0.001 | *** |  | -2.351 | 0.730 | -3.222 | 0.001 | ** |  | -2.756 | 0.785 | -3.510 | <0.001 | *** |
| Brown | nt | nt | nt | nt |  |  | -3.045 | 0.998 | -3.051 | 0.002 | ** |  | nt | nt | nt | nt |  |
| Red | nt | nt | nt | nt |  |  | -1.658 | 0.541 | -3.063 | 0.002 | ** |  | nt | nt | nt | nt |  |
| Yellow | nt | nt | nt | nt |  |  | -2.351 | 0.731 | -3.216 | 0.001 | ** |  | -1.236 | 0.457 | -2.703 | 0.007 | ** |
|  | *Agrilus hastulifer* | | | |  |  | *Agrilus laticornis* | | | |  |  | *Chrysobothris affinis* | | | |  |
| Vs. Green | EST | SE | z | P |  |  | EST | SE | z | P |  |  | EST | SE | z | P |  |
| Blue | -2.638 | 0.521 | -5.067 | <0.001 | *** |  | nt | nt | nt | nt |  |  | 1.946 | 1.005 | 1.937 | 0.053 | ● |
| Purple | nt | nt | nt | nt |  |  | nt | nt | nt | nt |  |  | 2.485 | 0.977 | 2.545 | 0.011 | * |
| Grey | -2.634 | 0.521 | -5.060 | <0.001 | *** |  | -3.644 | 1.139 | -3.201 | 0.001 | ** |  | 0.693 | 1.164 | 0.596 | 0.551 | ns |
| Black | nt | nt | nt | nt |  |  | -3.007 | 0.888 | -3.388 | <0.001 | *** |  | 1.099 | 1.092 | 1.006 | 0.314 | ns |
| Brown | nt | nt | nt | nt |  |  | nt | nt | nt | nt |  |  | 1.386 | 1.050 | 1.320 | 0.187 | ns |
| Red | nt | nt | nt | nt |  |  | nt | nt | nt | nt |  |  | 0.000 | 1.359 | 0.000 | 1.000 | ns |
| Yellow | -2.922 | 0.595 | -4.910 | <0.001 | *** |  | -1.320 | 0.558 | -2.366 | 0.018 | * |  | nt | nt | nt | nt |  |
|  | *Lamprodila mirifica* | | | |  |  |  |  |  |  |  |  |  |  |  |  |  |
| Vs. Green | EST | SE | z | P |  |  |  |  |  |  |  |  |  |  |  |  |  |
| Blue | -3.481 | 0.698 | -4.984 | <0.001 | *** |  |  |  |  |  |  |  |  |  |  |  |  |
| Purple | -4.174 | 0.933 | -4.476 | <0.001 | *** |  |  |  |  |  |  |  |  |  |  |  |  |
| Grey | nt | nt | nt | nt |  |  |  |  |  |  |  |  |  |  |  |  |  |
| Black | nt | nt | nt | nt |  |  |  |  |  |  |  |  |  |  |  |  |  |
| Brown | nt | nt | nt | nt |  |  |  |  |  |  |  |  |  |  |  |  |  |
| Red | nt | nt | nt | nt |  |  |  |  |  |  |  |  |  |  |  |  |  |
| Yellow | nt | nt | nt | nt |  |  |  |  |  |  |  |  |  |  |  |  |  |
