# Supplementary material for: Effect of Trap Color on Captures of Bark- and Wood-Boring Beetles (Coleoptera; Buprestidae and Scolytinae) and Associated Predators
Source: Insects. 2020 Oct 30;11(11):749. doi: 10.3390/insects11110749 (PMC7694114; doi:10.3390/insects11110749)
Supplement: Supplementary file 1 [file insects-11-00749-s001.zip › Compressed_Supplementary_files/Table_S4.docx]

**Table S4.** results of the GLMMs (i.e., contrasts vs black) testing the effect of trap color on bark and ambrosia (Scolytinae) beetles and checkered beetles (Cleridae). Model estimate (est), standard error (SE), z and P values are presented for each model. Colors that attracted a significantly different mean number of species or individuals than black traps are indicated in bold and with black asterisk/s or black circle based on the p-value: *** = P<0.001; ** = P<0.01; * P<0.05; ● = P<0.1.

| **Scolytinae** | | | | | | | | | | | | | | | | | | | | | | | |
| --- | --- | --- | --- | --- | --- | --- | --- | --- | --- | --- | --- | --- | --- | --- | --- | --- | --- | --- | --- | --- | --- | --- | --- |
|  | Species richness | | | |  |  | Abundance | | | |  |  | *Hylesinus oleiperda* | | | |  |  | *Scolytus multistriatus* | | | |  |
| Vs. Black | EST | SE | z | P |  |  | EST | SE | z | P |  |  | EST | SE | z | P |  |  | EST | SE | z | P |  |
| Brown | 0.013 | 0.159 | 0.084 | 0.933 | ns |  | -0.030 | 0.271 | -0.112 | 0.911 | ns |  | **-1.322** | **0.547** | **-2.417** | **0.016** | ***** |  | nt | nt | nt | nt |  |
| Red | -0.056 | 0.162 | -0.343 | 0.731 | ns |  | -0.304 | 0.274 | -1.109 | 0.267 | ns |  | -0.310 | 0.390 | -0.796 | 0.426 | ns |  | 0.916 | 1.175 | 0.780 | 0.435 | ns |
| Yellow | -0.279 | 0.171 | -1.633 | 0.102 | ns |  | **-0.857** | **0.278** | **-3.082** | **0.002** | ****** |  | **-0.916** | **0.472** | **-1.941** | **0.052** | **●** |  | -0.693 | 1.476 | -0.469 | 0.639 | ns |
| Green | -0.193 | 0.169 | -1.146 | 0.252 | ns |  | **-0.577** | **0.291** | **-1.987** | **0.047** | ***** |  | **-0.762** | **0.446** | **-1.708** | **0.088** | **●** |  | 0.693 | 1.196 | 0.580 | 0.562 | ns |
| Blue | 0.013 | 0.159 | 0.084 | 0.933 | ns |  | -0.195 | 0.276 | -0.705 | 0.481 | ns |  | **0.726** | **0.307** | **2.367** | **0.018** | ***** |  | **2.862** | **1.099** | **2.604** | **0.009** | ****** |
| Purple | 0.000 | 0.160 | 0.000 | 1.000 | ns |  | -0.134 | 0.275 | -0.486 | 0.627 | ns |  | **0.789** | **0.303** | **2.603** | **0.009** | ****** |  | 0.406 | 1.230 | 0.330 | 0.742 | ns |
| Grey | -0.114 | 0.164 | -0.698 | 0.485 | ns |  | -0.078 | 0.284 | -0.273 | 0.785 | ns |  | 0.182 | 0.341 | 0.535 | 0.592 | ns |  | **3.541** | **1.093** | **3.240** | **0.001** | ****** |
|  | *Xyleborinus saxesenii* | | | |  |  | *Xyleborus monographus* | | | |  |  | *Xylosandrus crassiusculus* | | | |  |  |  | | | |  |
| Vs. Black | EST | SE | z | P |  |  | EST | SE | z | P |  |  | EST | SE | z | P |  |  |  |  |  |  |  |
| Brown | -0.1243 | 0.2823 | -0.44 | 0.65974 | ns |  | **-1.120** | **0.450** | **-2.490** | **0.013** | ***** |  | 0.206 | 0.288 | 0.715 | 0.475 | ns |  |  |  |  |  |  |
| Red | -0.3424 | 0.2888 | -1.186 | 0.235789 | ns |  | -0.211 | 0.356 | -0.591 | 0.554 | ns |  | -0.164 | 0.293 | -0.559 | 0.576 | ns |  |  |  |  |  |  |
| **Yellow** | **-1.0951** | **0.2942** | **-3.722** | **0.000198** | ******* |  | **-2.513** | **0.760** | **-3.307** | **0.001** | ******* |  | **-0.771** | **0.300** | **-2.570** | **0.010** | ***** |  |  |  |  |  |  |
| **Green** | **-0.7418** | **0.3078** | **-2.41** | **0.015959** | ***** |  | **-1.599** | **0.527** | **-3.035** | **0.002** | ****** |  | -0.465 | 0.306 | -1.519 | 0.129 | ns |  |  |  |  |  |  |
| Blue | -0.310 | 0.289 | -1.070 | 0.285 | ns |  | **-0.984** | **0.433** | **-2.270** | **0.023** | ***** |  | **-0.490** | **0.298** | **-1.646** | **0.100** | **●** |  |  |  |  |  |  |
| Purple | -0.3069 | 0.2871 | -1.069 | 0.284979 | ns |  | -0.036 | 0.347 | -0.103 | 0.918 | ns |  | -0.286 | 0.294 | -0.971 | 0.331 | ns |  |  |  |  |  |  |
| **Grey** | **-0.609** | **0.2928** | **-2.08** | **0.037567** | ***** |  | **-1.035** | **0.435** | **-2.378** | **0.017** | ***** |  | **-0.523** | **0.295** | **-1.769** | **0.077** | **●** |  |  |  |  |  |  |
| **Cleridae** | | | | | | | | | | | | | | | | | | | | | | | |
|  | Species richness | | | |  |  | Abundance | | | |  |  | *Clerus mutillarius* | | | |  |  | *Thanasimus formicarius* | | | |  |
| Vs. Black | EST | SE | z | P |  |  | EST | SE | z | P |  |  | EST | SE | z | P |  |  | EST | SE | z | P |  |
| Brown | 0.140 | 0.279 | 0.501 | 0.617 | ns |  | **0.223** | **0.106** | **2.099** | **0.036** | ***** |  | **0.23** | **0.11** | **2.09** | **0.04** | ***** |  | -0.095 | 0.427 | -0.223 | 0.823 | ns |
| Red | 0.140 | 0.279 | 0.501 | 0.616 | ns |  | **0.229** | **0.107** | **2.138** | **0.033** | ***** |  | **0.24** | **0.11** | **2.19** | **0.03** | ***** |  | -0.452 | 0.469 | -0.963 | 0.336 | ns |
| Yellow | 0.095 | 0.280 | 0.340 | 0.734 | ns |  | **-0.636** | **0.120** | **-5.290** | **0.000** | ******* |  | **-0.65** | **0.13** | **-5.16** | **0.00** | ******* |  | **-1.705** | **0.737** | **-2.313** | **0.021** | ***** |
| Green | -0.051 | 0.300 | -0.171 | 0.864 | ns |  | **-0.349** | **0.114** | **-3.056** | **0.002** | ****** |  | **-0.33** | **0.12** | **-2.82** | **0.00** | ****** |  | **-1.012** | **0.568** | **-1.781** | **0.075** | **●** |
| Blue | -0.163 | 0.308 | -0.528 | 0.597 | ns |  | -0.169 | 0.112 | -1.518 | 0.129 | ns |  | -0.14 | 0.12 | -1.21 | 0.23 | ns |  | **-1.705** | **0.746** | **-2.285** | **0.022** | ***** |
| Purple | 0.000 | 0.299 | 0.000 | 1.000 | ns |  | 0.149 | 0.107 | 1.394 | 0.163 | ns |  | 0.16 | 0.11 | 1.47 | 0.14 | ns |  | -0.095 | 0.428 | -0.223 | 0.824 | ns |
| Grey | 0.000 | 0.296 | 0.000 | 1.000 | ns |  | **-0.263** | **0.113** | **-2.326** | **0.020** | ***** |  | **-0.26** | **0.12** | **-2.24** | **0.03** | ***** |  | 0.000 | 0.416 | 0.000 | 1.000 | ns |
